# Comparison of lidocaine bicarbonate with fentanyl and chloroprocaine for epidural anesthesia during cesarean section: a randomized, controlled, double-blind clinical trial

**DOI:** 10.3389/fphar.2024.1432918

**Published:** 2024-08-28

**Authors:** Jing Yu, Jingjing Liu, Chenran Wang, Congzhong Song, Gangming He, Chaohui Liu, Zhong Mei, Shaoqiang Huang

**Affiliations:** ^1^ Department of Anesthesia, Affiliated Zhejiang Xiaoshan Hospital of Hangzhou Normal University, Hangzhou, China; ^2^ Department of Anesthesia, Obstetrics and Gynecology Hospital of Fudan University, Shanghai, China

**Keywords:** cesarean section, chloroprocaine, epidural anesthesia, fentanyl, lidocaine bicarbonate

## Abstract

Chloroprocaine and lidocaine bicarbonate are commonly used for epidural anesthesia because of their rapid onset, particularly in the case of conversion from epidural labor analgesia to emergency cesarean section. However, it is unclear whether lidocaine bicarbonate combined with fentanyl has an advantage over chloroprocaine alone in emergency cesarean section. In this study, 102 women who underwent elective cesarean section received 15 mL 3% chloroprocaine and 1 mL saline (CP group) or 15 mL 1.73% lidocaine bicarbonate and 1 mL fentanyl 50 μg (LF group) for epidural anesthesia. Nociceptive block level was assessed by pinprick and recorded every minute. The primary outcome was the onset time to T6 block. The median onset time to T6 analgesia was 10 [10, 10] min in the CP group and 10 [7, 10] min in the LF group (COX model for CP *versus* LF, HR 0.47, 95% CI 0.23–0.95, *p* = 0.035). The median onset time to T8 analgesia was 7 [5, 9] min in CP group and 5 [4, 7] min in LF group (COX model for CP *versus* LF, HR 0.61, 95% CI 0.39–0.95, *p* = 0.027). The proportion of hypotension episodes occurring before delivery in LF group was lower than that in CP group (*p* = 0.011). The incidence of block level ≥ T4 after supplemental dosing in the LF group was lower than that in the CP group (*p* = 0.031). Compared with 3% chloroprocaine, 1.73% lidocaine bicarbonate combined with fentanyl 50 μg has a slightly faster onset time and less hypotension in epidural anesthesia for cesarean section.

**Clinical Trial Registration:** http://www.chictr.org.cn/index.html, identifier ChiCTR2200056180.

## Introduction

Epidural block is the most widely used method for labor analgesia. It provides safe and reliable analgesia to the parturient during labor and can also be extended to surgical anesthesia for emergency cesarean section (CS) when a fast-acting epidural regimen is required.

Chloroprocaine is a quick-acting, short-lasting, amino ester local anesthetic, and rarely crosses the placental barrier. It is commonly used for cesarean epidural anaesthesia and often recommended for emergency cases because of its rapid onset (Sharawi et al., 2021). Lidocaine bicarbonate is made of lidocaine hydrochloride and sodium bicarbonate under carbon dioxide saturation; it has more uncharged base form than lidocaine hydrochloride which can promote the diffusion and uptake of lidocaine, thereby accelerating the onset and enhancing the blocking effect (Lechat et al., 2023). In addition, previous studies have shown that the addition of lipophilic opioids, such as fentanyl, to local anesthetic solutions could shorten the latency and enhance the potency of local anesthetics in epidural block (Sng et al., 2008; Hillyard et al., 2011; Sanders et al., 2004; Nanji and Carvalho, 2020). Therefore, lidocaine bicarbonate is often administered along with fentanyl for epidural anaesthesia.

Although both chloroprocaine and lidocaine bicarbonate with fentanyl have rapid onsets, few studies have directly compared these two methods in epidural anesthesia for CS. Recently, Sharawi et al. (2021) simulated the conversion of epidural labor analgesia to surgical anesthesia and compared onset times of 3% chloroprocaine with the mixture of 2% lidocaine, 150 μg of epinephrine, 2 mL of 8.4% bicarbonate, and 100 μg fentanyl (LEBF). They concluded that both solutions provide rapid onset of anesthesia when used to extend the low-dose epidural sensory block to surgical anesthesia, and the noninferiority of chloroprocaine to LEBF could not be demonstrated. However, LEBF preparation is time-consuming and cannot be completely standardized.

Commercial 1.73% lidocaine bicarbonate solution is available and convenient for emergency anesthesia. However, no studies have directly compared the characteristics of this commercially available lidocaine bicarbonate solution with those of chloroprocaine for epidural anesthesia.

In this study, the characteristics of 3% chloroprocaine and 1.73% lidocaine bicarbonate combined with 50 μg fentanyl for epidural anesthesia were compared in CS. Our primary outcome was the onset time to T6 block. Secondary outcomes included onset time to T8 block, highest sensory block level, proportion of epidural supplemental dose, motor block, proportion of esketamine usage, and incidence of adverse reactions. We hypothesized that 1.73% lidocaine bicarbonate and fentanyl would have a faster onset time than 3% chloroprocaine in epidural anesthesia for CS.

## Materials and methods

### Study design and participants

**FIGURE 1:**
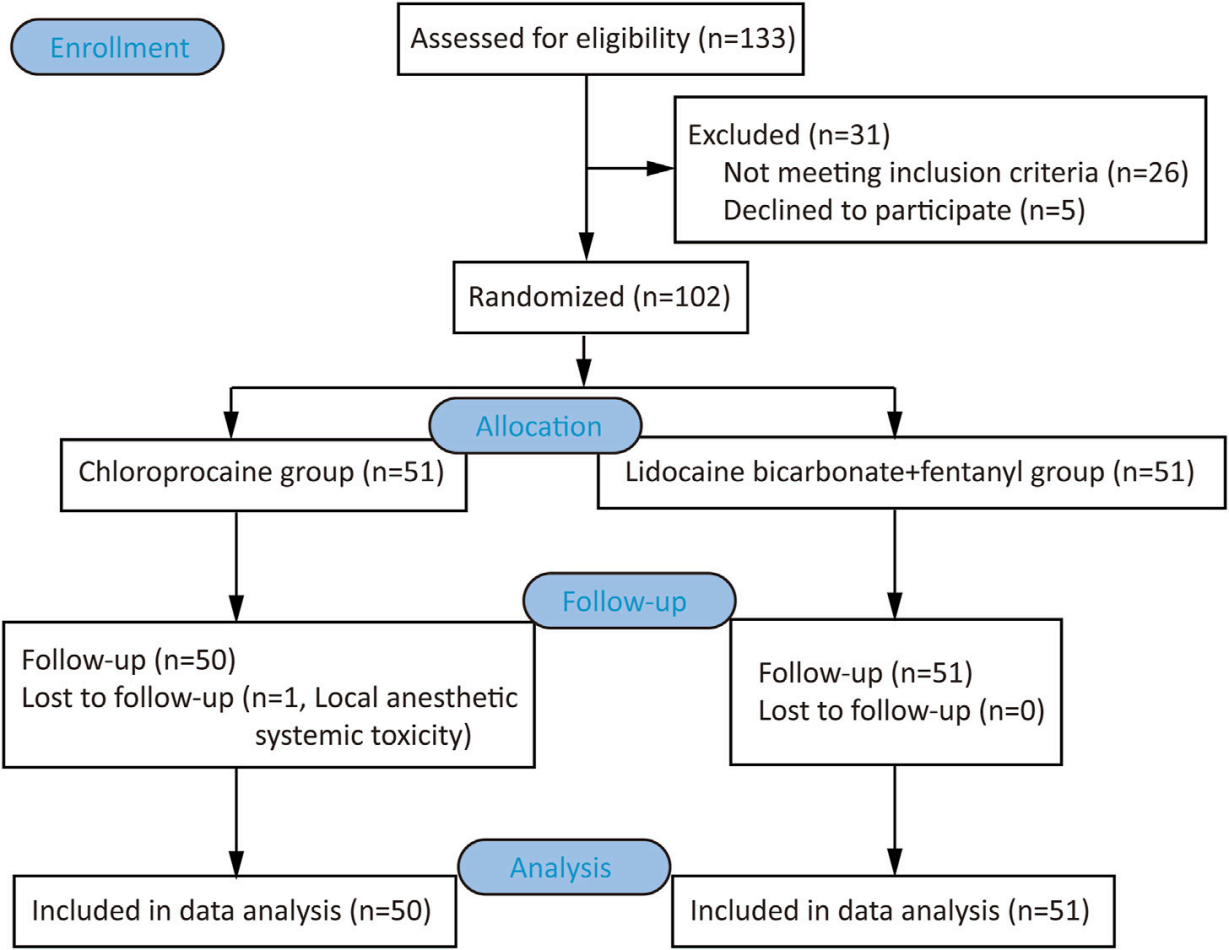
Flow chart of the clinical trial.

We conducted a prospective, double-blind, and single-centre RCT at Affiliated Zhejiang Xiaoshan Hospital of Hangzhou Normal University located in Hangzhou, China. Ethical approval for this study (Ethical Committee No. KL2022031) was provided by the Ethics Committee of Zhejiang Xiaoshan Hospital, Hangzhou, China (Chairman Prof. Guojun Jiang) on 21 January 2022. And it has been registered at the Chinese Clinical Trial Registry (https://www.chictr.org.cn/showproj.html?proj=151199, registration number: ChiCTR2200056180, principal investigator: Jing Yu, date of registration: 1 February 2022) before participant enrollment. This article adheres to the applicable Consolidated Standards of Reporting Trials (CONSORT) guidelines. Written informed consents were obtained from all participants. The study flowchart is shown in Figure 1.

### Flow chart of the clinical trial

Full-term parturients who underwent elective CS at Xiaoshan Hospital from February 8 to 8 May 2022, were screened for inclusion in this study. The inclusion criteria were ASA II, age 18–40 years old, height 150–170 cm, and weight ≤100 kg. Participants were excluded if they refused participation, or had allergies to study drugs, spinal deformity, twin or multiple pregnancies, contraindications to neuraxial block (e.g., coagulopathy, infection of puncture sites) and gestational diseases (e.g., preeclampsia, gestational diabetes mellitus). Participants were withdrawn from the study for epidural puncture failure, intravascular and subarachnoid catheters, unilateral block, or less than T8 block 15 min after anesthetic induction.

#### Randomization and blinding

A random number table was generated using SPSS (Version 26.0, SPSS Inc., Chicago, Illinois, USA) by Chenran Wang who was not involved in the recruitment or clinical management of the participants. All participants were assigned to either the CP group or the LF group. The CP group received an anesthetic induction dose of 15 mL 3% chloroprocaine (No.2020034, Haisi Pharmaceutical Co., Ltd., Jincheng, Shanxi Province, China). The LF group received 15 mL 1.73% lidocaine bicarbonate (No. 2006013, Jichuan Pharmaceutical Co., LTD., Taizhou, Jiangsu Province, China) and 50 μg fentanyl (No. 01D09081A2, Renfu Pharmaceutical Co., LTD, Yichang, Hubei Province, China). For allocation concealment, we used numbered, sealed, and opaque envelopes. An anesthesia nurse, not involved in the observation or data collection, was responsible for opening the sealed envelope and preparing the study medications. The anesthesiologists, researchers assessing outcomes, data collectors, and statisticians performing the analysis were all blinded to the group allocations.

To ensure blinding, three syringes containing the infusions were prepared in advance by the anesthesia nurse. Each participant received the first syringe containing 15 mL of the assigned local anesthetic for induction. The second syringe, labeled “Adjuvant”, contained 1 mL of 50 μg fentanyl for the LF group or 1 mL of normal saline for the CP group. The third syringe, labeled “Supplement”, contained 5 mL of the respective local anesthetic: 3% chloroprocaine for the CP group and 1.73% lidocaine bicarbonate for the LF group.

#### Research process

Parturients fasted for 8 hours and did not drink for 2 hours. No parturient received preoperative drug treatment. After entering the operating room, all participants received 500 mL lactated Ringer’s solution and underwent routine monitoring, including noninvasive blood pressure, electrocardiogram, and oxygen saturation.

Standard epidural anesthesia was administered by attending anesthesiologists who were blinded to the group allocations. The epidural puncture was performed at the L2–3 intervertebral space, and an epidural catheter was inserted 3–4 cm into the epidural space. Initially, according to randomization, a test dose was administered through the catheter: 3 mL of 3% chloroprocaine for the CP group and 3 mL of 1.73% lidocaine bicarbonate for the LF group. Immediately following this, the “Adjuvant” was injected. After 3 minutes of observation, the remaining 12 mL of local anesthetic from the first syringe was injected into the epidural space over a period of 30–40 s. The completion of this injection was defined as Time 0.

Sensory blocking was measured by two researchers. The needle was pressed onto the skin at 1-min intervals for the first 10 minutes, gradually moving from the thigh to the chest. The participant was asked “Tell me when you feel something sharp touching your skin”. When the parturient experienced pain at the T5 level, it was defined as T6 analgesia, which is suitable for CS. If the analgesia level was less than T6 more than 10 minutes following induction, the “Supplement” syringe was administered. If the bilateral block was still below T8 5 minutes later, the participant was withdrawn from the study and anesthesia method was adjusted for the surgery. If the block level exceeded T8 but not T6, esketamine 0.3 mg kg^-1^ was administered at the discretion of the anesthesiologist to prevent intraoperative pain. If the block level exceeded T6, but the parturient still felt pain during the operation, esketamine 0.15 mg kg^-1^ would be administered intravenously. All parturients received an additional 5 mL 0.5% ropivacaine and 1 mL hydromorphone (0.4 mg) 30 min after anesthetic induction for postoperative analgesia.

For intraoperative hypotension, defined as a reduction in systolic blood pressure ≥20% of the baseline value or <90 mmHg, 50 µg phenylephrine was administered intravenously as necessary. For bradycardia, defined as a heart rate <50 beats/min, 0.5 mg atropine was administered intravenously. Satisfaction with anesthesia (very satisfied, satisfied, acceptable, or unacceptable) was evaluated before parturients were transferred to the ward.

### Sample size

In our preliminary experiment, the mean time to T6 block in the LF group (15 mL in full-term parturients was 9.54 (2.76) minutes. We defined 2 minutes as a clinically significant difference based on previous studies (Gaiser et al., 1998). According to the bilateral α = 0.05, β = 0.10, the necessary sample size was calculated to be 42 using PASS (Version 15.0.5. NCSS, LLC: Kaysville; 2017.). To allow for 20% drop-out rate, finally 51 participants in each group was included.

### Statistical analysis

SPSS version 26.0 was used for data analysis. After a Kolmogorov-Smirnov test assessing the normality of data distribution, data were shown as mean ± standard deviation (SD) or median (interquartile range [IQR]), as appropriate. Quantitative variables were compared between groups using unpaired Student’s t*-*test when data were normally distributed, or the Mann-Whitney *U* test when data were not normally distributed. Qualitative variables were expressed as frequency (*n*) and *n*/total *N* (%). The Chi square test or Fisher’s exact probability methods were used for between-group comparisons. The onset times to T6 and T8 blocks in both groups are shown as Kaplan-Meier survival curves. Hazard ratios (HR) for the two groups were calculated using the Cox Proportional Hazard Regression Model. *p* < 0.05 was considered statistically different.

## Results

A total of 133 women were screened for eligibility. Thirty-one participants were excluded because of declining to participate or not meeting the inclusion criteria, and one participant in CP group was withdrawn because local anesthetic systemic toxicity occurred to her after injecting the test dose (Anesthetist administered general anesthesia for this parturient. Maternal and neonatal outcomes are well). Finally, 101 parturients (50 in the CP group and 51 in the LF group) were included (Figure 1). There were no differences in maternal age, height, weight, gestational age, parity, number of cesarean sections, or operation time between the two groups (Table 1).

**TABLE 1:** Baseline characteristics of the parturients.

	Group CP (n = 50)	Group LF (n = 51)	*p-v*alue
Age (year)	29 [26, 31]	30 [26, 32]	0.176^*^
Height (cm)	159 [155, 162]	160 [156, 163]	0.147^a^
Weight (kg)	68 ± 10	71 ± 8	0.054^*^
Gestational age (day)	274 ± 5	273 ± 6	0.650^*^
Parity (pri-/multi-)	24/26	20/31	0.373^b^
Number of cesarean section (0/1/2)	25/21/4	24/25/2	0.592^b^
Surgical time (min)	36 ± 10	39 ± 11	0.397^*^

Data are shown as Mean ± Standard deviation or Median [25th to 75th quartiles]. Group CP, 3% chloroprocaine; Group LF, 1.73% lidocaine and 50 μg fentanyl.^*^The independent samples *t*-test.

^a^ The Mann-Whitney *U* test.

^b^ The Chi-square test or Fisher exact test.

**FIGURE 2:**
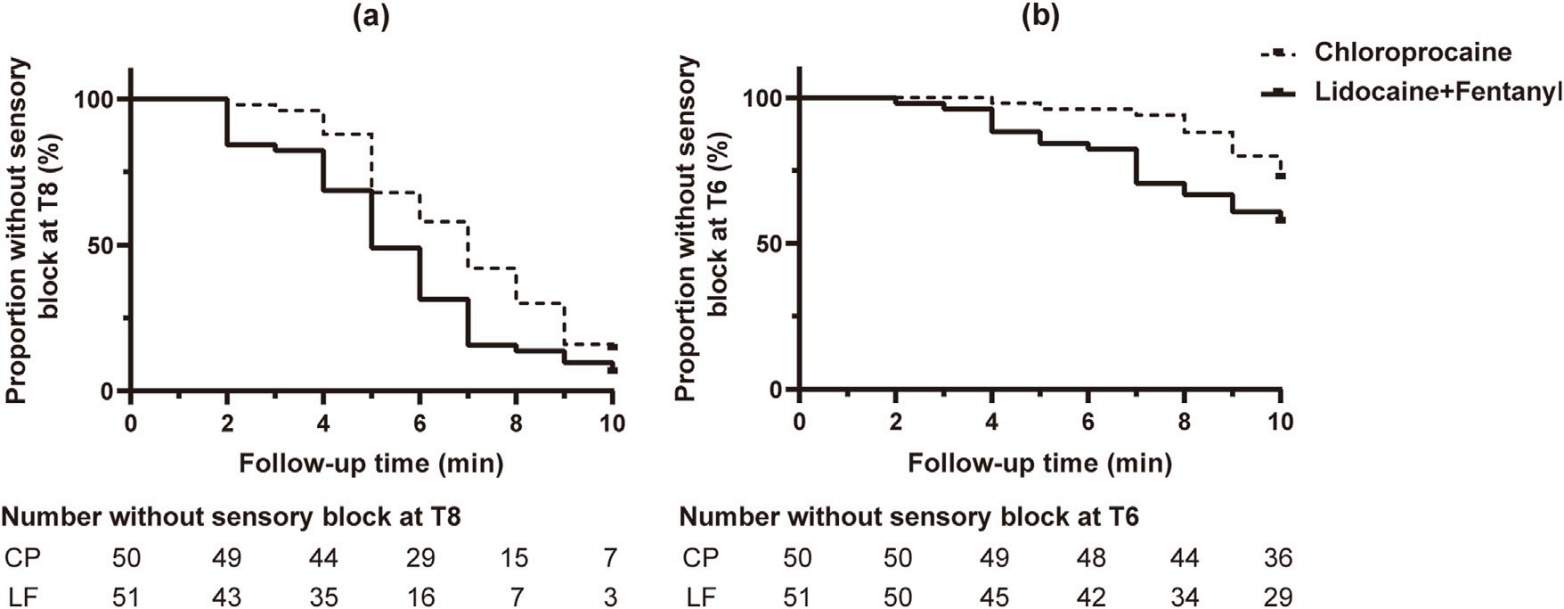
Kaplan-Meier survival curve for time to onset of sensory block to pinprick. **(A)** Block at T8. **(B)** Block at T6. More parturient in group LF reached T8 and T6 blockade than those of group CP in the Cox model (*p* = 0.027, *p* = 0.035). Censored observations are shown as black dots in bold.

We considered 10 minutes after induction as the end point for the initial dose and constructed Kaplan-Meier survival curves to show the onset time to T6 and T8 analgesia in both groups (Figure 2). The median onset time to T6 analgesia was 10 [10, 10] min in the CP group and 10 [7, 10] min in the LF group. On the Cox model (concomitant variables: history of cesarean section, 35 years old, weight 70 kg, height 160 cm) for CP *versus* LF, the HR was 0.47, 95% CI 0.23–0.95, *p* = 0.035. For T8 analgesia, the median onset time was 7 [5, 9] min in CP group and 5 [4, 7] min in LF group. On the Cox model, for CP *versus* LF, the HR was 0.61, 95% CI 0.39–0.95, *p* = 0.027). These data indicate that the time to T6 and T8 analgesia onset was shorter in the LF group than in the CP group (Figure 2; Table 2).

**TABLE 2:** Summary of trial outcomes of parturients.

	Group CP (n = 50)	Group LF (n = 51)	*p-v*alue
Successful T6 block at 10 min, n (%)	14 (28%)	22 (43%)	0.112^a^
Successful T8 block at 10 min, n (%)	43 (86%)	48 (94%)	0.200^a^
Onset of T6 analgesia (min)	10 [10, 10]	10 [7, 10]	0.035^b^
Onset of T8 analgesia (min)	7 [5, 9]	5 [4, 7]	0.027^b^
Highest sensory block level (>T4/T4-6/<T6)	10/33/7	8/40/3	0.289^a^
Epidural supplemental dose during anesthesia induction	36/50 (72%)	28/51 (55%)	0.075^a^
Incidence of >T4 block after epidural supplemental dose	6/36 (17%)	0/28 (0%)	0.031^a^
Modified Bromage score (0/1/2/3)			
at 5 min	23/24/3/0	25/22/4/0	0.864^a^
at 10 min	0/20/30/0	2/26/22/1	0.122^a^
Remedical use of esketamine	13 (26%)	10 (20%)	0.444^a^
Maternal satisfaction (very satisfied/satisfied/acceptable/unacceptable)	50/0/0/0	50/0/1/0	0.500^a^

Data are shown as Median [25th to 75th quartiles] or number (proportion). Group CP, 3% chloroprocaine; Group LF, 1.73% lidocaine and 50 μg fentanyl.

^a^ The; Chi-square test or Fisher exact test.

^b^ The Cox Proportional Hazard Regression Model. For onset of T6 analgesia, the Hazard Ratio is 0.47 (95% CI, 0.23–0.95). For onset of T8 analgesia, the Hazard Ratio is 0.61 (95%CI, 0.39–0.95).

The proportion requiring a supplemental local anesthetic dose during anesthesia induction was 55% in the LF group and 72% in the CP group, which was not statistically different (*p* = 0.075). For parturients who received supplemental local anesthetics, 0/28 (0%) parturients in the LF group, while 6/36 (17%) parturients in the CP group had a block level higher than T4 (*p* = 0.031). These data indicated that parturients who received supplemental local anesthetics in the CP group were more likely to have high-level block than those in the LF group (Table 2).

There was no difference in the proportion of esketamine usage between the CP (13/50, 26%) and LF (10/51, 20%) groups. Among these, five parturients in each group were for intraoperative traction pain, although the analgesia level reached T6 or above. In addition, three cases in each group had analgesia levels less than T6 15 min after induction. Because the parturients did not have any intraoperative pain, they did not receive esketamine, and the operation was successful. All women treated with esketamine completed the operation successfully without adverse effects. Except one parturient in the LF group thought the anesthesia was not satisfying because of nausea and vomiting, and all other parturient were very satisfied or satisfied with anesthesia (Table 2).

All 16 (100%) episodes of hypotension in the CP group occurred before fetal delivery, while 8/13 (62%) episodes of hypotension in the LF group occurred before fetal delivery (*p* = 0.011). There were no significant differences in other outcomes between the two groups (Table 3).

**TABLE 3:** Maternal adverse reactions and neonatal apgar scores.

	Group CP (n = 50)	Group LF (n = 51)	*p-v*alue
Hypotension	16 (32%)	13 (26%)	0.470^a^
Time to occurrence of hypotension (min)	10 ± 6	20 ± 17	0.068^b^
The block level when hypotension occurred	T7 [T6, T10]	T6 [T4, T8]	0.276^a^
Hypotension before delivery	16 (32%)	8 (16%)	0.054^a^
Hypotension before delivery/All hypotension cases	16/16 (100%)	8/13 (62%)	0.011^a^
Nausea and vomiting	2 (4%)	4 (8%)	0.692^a^
Pruritus	2 (4%)	2 (4%)	1.000^a^
Neonatal Apgar Scores			
at 1 min	10 ± 0.7	10 ± 0.0	0.151^b^
at 5 min	10 ± 0.0	10 ± 0.0	1.000^b^

Data are shown as Mean ± Standard deviation or Median [25th to 75th quartiles] or number (proportion). Group CP, 3% chloroprocaine; Group LF, 1.73% lidocaine and 50 μg fentanyl.

^b^ The independent samples *t*-test.

^a^ The Chi-square test or Fisher exact test.

## Discussion

In this study, we recruited women undergoing elective cesarean section to compare the onset time and block characteristics of 1.73% lidocaine carbonate with fentanyl and 3% chloroprocaine for epidural anesthesia. The onset to T6 and T8 blocks within 10 minutes was faster in the LF group than in the CP group. More than half of the participants in both groups received additional supplemental doses during anesthesia induction, indicating that 15 mL 3% chloroprocaine or 1.73% lidocaine bicarbonate combined with 50 µg fentanyl was insufficient for emergency CS in most parturients.

In clinical practice, rapid and adequate anesthesia is required for conversion of vaginal delivery to cesarean section due to emergency situations such as fetal intrauterine distress. For women who have already received epidural analgesia, the analgesia block can be extended for surgical anaesthesia, avoiding the risks associated with re-puncture and general anaesthesia (Hillyard et al., 2011; Ituk and Wong, 2020). Therefore, it is important to choose a rapid-onset local anesthetic to accelerate epidural anaesthesia.

Recently, a Bayesian Network meta-analysis confirmed that chloroprocaine and alkalized lidocaine are the fastest acting local anesthetics for caesarean epidural anaesthesia; however, they did not compare chloroprocaine and alkalized lidocaine directly (Reschke et al., 2020). Sharawi et al. (2021) first compared alkalized lidocaine and chloroprocaine for epidural anaesthesia in CS, while the alkalized lidocaine in their study was prepared prior to use, which is not appropriate in emergency situations as it slows the time to administration. Additionally, their study was designed as a non-inferiority trial. Commercial lidocaine carbonate solution has more advantages in this regard. Our study is the first superiority trial comparing commercial lidocaine carbonate and chloroprocaine for cesarean delivery under epidural anesthesia.

In our study, fentanyl was combined with lidocaine bicarbonate, this is due to that lipophilic opioids in either epidural (Hillyard et al., 2011; Bachmann-Mennenga et al., 2005) or spinal anaesthesia (Hamber and Viscomi, 1999; Uppal et al., 2020) can accelerate the onset of local anesthetics, enhance the effect, and prolong the analgesia time. Therefore, it has become routine to add low-dose opioids to local anesthetics for neuraxial anaesthesia for CS (Practice Guidelines for Obstetric Anesthesia, 2016; American College of Obstetricians and Gynecologists’ Committee on Practice Bulletins—Obstetrics, 2019). Nonetheless, some studies have found that chloroprocaine can antagonize the analgesic effect of epidural opioids (including morphine and fentanyl) (Camann et al., 1990; Sc et al., 1990; Eisenach et al., 1991; Karambelkar and Ramanathan, 1997; Johnson et al., 1991). Therefore, we did not combine chloroprocaine with fentanyl in this study.

In England, T4 block of cold sensation was considered as the anesthetic level required for CS (Bourne et al., 1997; Allen et al., 2022). The loss of T6 touch also enables most parturients to undergo CS without pain (Russell, 2004). The level of anaesthesia to cold sensation is, on average, two spinal segments higher than the level of pinprick (Camorcia and Capogna, 2006). Thus, we used the loss of T6 nociception as the satisfactory block level for CS, which was equivalent to the anaesthesia of T4 cold sensation blockade. As pain sensation from pelvic organs enter the spinal cord at T10–L1, T8 pain loss is the most basic requirement for emergency CS (Quan et al., 2015). Therefore, we evaluated the time to T6 and T8 analgesia to compare the onset time of the two local anesthetic. Moreover, we used esketamine to assist anaesthesia, which has stronger analgesic potency, faster recovery, and fewer mental adverse reactions (Suppa et al., 2012; Peltoniemi et al., 2016).This seems to indicate that when the caesarean section is very urgent, auxiliary application of esketamine to start the operation as soon as possible after reaching T8 block may be a compromise solution. The incidence of ≥ T4 block after additive local anesthetics in the LF group was lower than that in the CP group. This suggests that compared to chloroprocaine, it is easier to predict the block level at an early stage using lidocaine bicarbonate and fentanyl.

The proportion of participant who have at least one hypotensive episode before delivery in the LF group was lower than that in the group CP, but the difference was not statistically significant (15.7% *versus* 32.0%, *p* = 0.054). However, among all episodes of hypotension, the proportion of hypotension before delivery was significantly lower in the LF group than that in the CP group (61.5% vs 100%, *p* = 0.011). A short period of hypotension after neuraxial anaesthesia before delivery does not endanger the mother, but may lead to fetal and neonatal asphyxia. Some studies have found that the degree and duration of hypotension after neuraxial anaesthesia during CS is associated with transient tachypnea in newborns (Singh et al., 2019). Therefore, the relatively high incidence of pre-delivery hypotension after epidural anaesthesia using chloroprocaine is worthy of attention.

In this study, the initial induction dose of local anesthetics in both groups was 15 mL. However, more than half of the women in both groups had a block level below T6 after 10 min of administration, requiring an additional 5 mL supplemental dose. This suggests that an induction dose of 15 mL is not sufficient for an emergency CS. Given that the majority of women in this study received 20 mL of local anesthetics and the overall low incidence of hypotension, 20 mL of 1.73% lidocaine carbonate or 3% chloroprocaine may be appropriate as an induction dose for epidural anesthesia in emergency CS, although further studies are needed to confirm this.

This study had some limitations. First, we did not use ultrasound guidance to identify the intervertebral space for epidural puncture. However, the probability of occurrence of this bias was similar between the two groups because of randomization. Second, the initial induction dose of local anesthetics used was 15 mL, which is relatively small for emergency CS. In future trials, a larger volume of the induction dose, such as 20 mL, should be considered. Third, we did not simulate a situation in which women had already received a low concentration of local anesthetics when they were converted from labor analgesia to CS anaesthesia. We aimed to investigate which of the two regimens was faster and more reliable for epidural block. Thus, none of the women had previously received any other treatments.

In summary, compared with 3% chloroprocaine, 1.73% lidocaine bicarbonate combined with fentanyl 50 μg in epidural anesthesia for cesarean section has a slight advantage in terms of the onset time and hypotension incidence. This indicated that lidocaine bicarbonate combined with fentanyl may be a better choice when labor analgesia is converted to emergency cesarean section anesthesia. To achieve a satisfactory block level as soon as possible after a single dose, the anesthetic induction dose of both local anesthetics should be greater than 15 mL.

## Data Availability

The raw data supporting the conclusions of this article will be made available by the authors, without undue reservation.
